# Phosphorus-Rich Ruthenium Phosphide Embedded on a 3D Porous Dual-Doped Graphitic Carbon for Hydrogen Evolution Reaction

**DOI:** 10.3390/nano12203597

**Published:** 2022-10-13

**Authors:** Aicha Anouar, Antonio Doménech-Carbó, Hermenegildo Garcia

**Affiliations:** 1 Engineering Division, Euromed Research Institute, EuroMed University of Fes (UEMF), Route de Meknes, Rond-Point de Bensouda, Fès 30070, Morocco; 2 Departamento de Química (UPV), Instituto de Tecnología Química (CSIC-UPV), Universitat Politècnica de València, Av. de los Naranjos s/n, 46022 Valencia, Spain; 3 Departament de Química Analítica, Universitat de València, Dr. Moliner 50, Burjassot, 46100 Valencia, Spain

**Keywords:** ruthenium electrocatalysts, metal phosphides, ruthenium diphosphide, dual-doped graphitic carbon, hydrogen evolution reaction

## Abstract

Metal phosphides have recently emerged as promising electrocatalysts for hydrogen evolution reaction (HER). Herein, we report the synthesis of ruthenium diphosphide embedded on a dual-doped graphitic carbon by pyrolyzing chitosan beads impregnated with ruthenium chloride and phosphorus pentoxide. The as-synthesized RuP_2_@N-P-C displays a good electrocatalytic activity in acidic, neutral and alkaline media. We show that the HER activity of the electrocatalyst can be tuned by varying the concentration of Li^+^ cations. Co-diffusion effects on H^+^ exerted by Li^+^ on HER in the porous carbon matrix have been observed.

## 1. Introduction

With the exhaustion of fossil fuels and ongoing global warming, referring to clean and sustainable energy resources is of paramount importance [1,2]. Hydrogen (H_2_), a carbon-free and clean energy is regarded as an attractive energy vector for transportation and for the chemical storage of green electricity [3,4]. The current production of hydrogen by steam reforming of natural gas consumes fossil fuels and emits equivalent amounts of carbon dioxide (CO_2_) contributing to the greenhouse effect [5,6]. Implementation of the industrial, large scale hydrogen production by electrocatalytic water splitting with renewable electricity is set to overcome these issues as it is a carbon-zero process allowing for the production of green hydrogen [7].

To this date, Pt-based electrocatalysts constitute the most active catalysts for hydrogen evolution reaction (HER) [8]. Unfortunately, however, the towering cost and scarcity of this element is one of the bottlenecks limiting the massive production of green hydrogen [9]. Most of the studies focusing on the remarkable activity of Pt are carried out in acidic media, yet Pt-based electrocatalysts display about two orders of magnitude lowered activity in alkaline media due to sluggish kinetics [10]. Ruthenium (Ru), a relatively cheaper metal has demonstrated respectable activity in HER with minute overpotentials and extremely low on-set potentials [11,12,13] ascribed to similar metal-hydrogen bonding to that of Pt and decreased water activation barrier [14]. To boost the HER activity, a modulation of the adsorption of reaction intermediates (hydrogen) to the active sites of the electrocatalysts is necessary. For Ru-based electrocatalysts, a strong adsorption of H on Ru surface is generally observed leading to a poor HER activity [15].

As a new class of materials, metal phosphides (MPs) have recently attracted great attention as electrocatalysts for HER and organic molecule electro-oxidation reactions due to their low cost and high activities [16,17,18]. Depending on the phosphorus content, MPs exhibit different activities in HER [19]. Compared to metal (M) rich MPs, phosphorus-rich MPs possess lower conductivities due to abundant P-P bonds and M-P bonds and lower density of M-M bonds [19]. Accordingly, RuP nanoparticles demonstrated superior electrocatalytic activity compared to RuP_2_ due to the abundance of electrocatalytic active sites and better conductivity [20]. One of the means to enhance the conductivity of P-rich MPs is to support them on carbon materials such as graphene and activated carbons [21]. The support generally exposes more active sites and boosts the electrical conductivity facilitating electron transfer [22]. Most of the strategies used for the synthesis of RuP_x_ supported on carbon matrices rely on the use of phosphines, or sodium hypophosphite and are generally not straightforward since several steps are needed to carry out phosphorization [23,24].

Biopolymers such as chitosan (CS) and sodium alginate have the ability to be shaped into three dimensional nanofibrillated microspheres thanks to their pH switchable solubility [25]. The hydrogels resulting from precipitation of these biopolymers in alkaline (CS) or acidic (alginic acid) aqueous solutions, when dehydrated with ethanol, can be dried using CO_2_ supercritical conditions and thus allow the obtainment of aerogels with a large surface area and open macroporous network [26]. Pyrolysis of aerogels has been greatly explored to prepare porous graphitic catalysts with intrinsic defects [27,28,29] on which metallic nanoparticles are grown in situ. Since CS and alginic acid based microspheres have excellent abilities to adsorb phosphates [30,31], P-doped or N,P-dual doped porous graphitic carbon could be easily synthesized through pyrolysis of biopolymers on which phosphates are adsorbed or grafted [21,32].

Recently, a phosphate ester obtained from dissolution of phosphorus pentoxide (P_2_O_5_) in methanol was revealed as an excellent precursor to prepare noncovalent phosphorylated graphene oxide and the resulting material displayed improved hole transport in polymer solar cells [33]. Inspired by this approach, we used P_2_O_5_ dissolved in ethanol as P source to grow MPs in situ during pyrolysis of metal containing CS microspheres. The strategy herein reported allows the synthesis of RuP_2_ nanoparticles supported in highly porous N,P-codoped graphitic carbon (RuP_2_@N-P-C). The electrocatalytic effect exerted by RuP_2_ nanoparticles embedded in nitrogen and phosphorus dual doped graphitic carbon on the hydrogen evolution reaction (HER) was studied at acidic, basic and neutral pHs using usual protocols [34,35,36,37]. In acidic media, the catalytic effect of RuP_2_@N-P-C was significantly increased in the presence of Li^+^ ions in the electrolyte. This effect, also known in literature, but of unclear explanation [38,39,40], is studied here focusing the attention on co-diffusion H^+^-Li^+^ effects based on voltammetric and electrochemical impedance spectroscopy (EIS) data. 

## 2. Materials and Methods

### 2.1. Synthesis of RuP_2_@N-P-C

CS MMW (1000 mg; ACROS) was dissolved in 50 mL of 1.25 vol% acetic acid solution. A complete dissolution was obtained after 21 h of stirring at room temperature. CS beads were formed by dropping the solution through a 0.8 mm syringe into 4 M NaOH solution. CS beads were kept in the alkaline solution for 3 h followed by extensive washings until neutral pH. Forty-two g of the hydrogels obtained were then immersed into a 3.7 mM aqueous RuCl_3_ solution for 24 h. The Ru-containing alcogels were obtained by exchanging water with ethanol at increasing ethanol/water ratios (10/90; 30/70; 50/50; 70/30; 90/10; and 100% ethanol). Impregnation of Ru containing alcogels with phosphate was carried out by immersing for 24 h the alcogels in a solution of 700 mg of phosphorus pentoxide (P_2_O_5_) dissolved in 100 mL of ethanol. Dry Ru-phosphate CS aerogels were obtained using an automated CO_2_ critical point dryer. The aerogels were pyrolyzed under Ar atmosphere at 5 °C/min until reaching a temperature of 900 °C that was maintained for 1 h. A content of Ru and P of 6.73% and 8.93%, respectively was determined by ICP-OES by dispersing 8 mg of RuP_2_ @N-P-C in 8 mL of *aqua regia* and heating at 100 °C for 24 h to completely dissolve Ru and P.

### 2.2. Characterization

Chemical elemental analysis of the electrocatalyst was determined by combustion chemical analysis using a CHNS FISONS elemental analyzer. Specific surface area of RuP_2_@N-P-C was determined by N_2_ adsorption isotherms using a Micromeritics 2010 instrument. Field Emission Scanning Electron Microscopy (FESEM) images were acquired using a ZEISS ULTRA 55 microscope equipped with an X-ray detector (EDS). Scanning Transmission Electron Microscopy (STEM) images were acquired using a JEOL JEM 2100F Field Emission Transmission Electron Microscope of 200 kV equipped with an X-ray detector. X-Ray diffraction patterns were recorded using a Cubix Pro PANanalytical diffractometer. Raman spectra were recorded using a 514 nm laser excitation on a Renishaw Raman spectrophotometer equipped with a LEICA microscope. XP spectra were measured on a SPECS spectrometer using a monochromatic X-Ray source (Al and Mg) operating at 200 W.

### 2.3. Electrochemical Measurements

Electrochemical measurements were performed in a conventional three-electrode electrochemical cell using an Ag/AgCl (3 M NaCl) reference electrode and a Pt wire auxiliary electrode. The working electrode was a catalyst-modified glassy carbon electrode (BAS, MF 2012, geometrical area 0.071 cm^2^, GCE). Voltammetric and EIS experiments were carried out with a CH 660I potentiostatic device (Cambria Scientific, Llwynhendy, Llanelli, Wales, UK). EIS measurements were carried out by superimposing different bias potentials to a sinusoidal excitation of 5 mV peak-to-peak amplitude in the 0.1 to 10^5^ Hz frequency range. Air-saturated 0.5 M H_2_SO_4_ and 1.0 M KOH aqueous solutions were used as electrolytes. The modification of the GCEs was initiated by polishing mechanically the bare GCE with 0.5 μm diamond down to 0.05 μm alumina slurry to obtain a mirror-like surface and then washed with Milli-Q water, ethanol and acetone and allowed to dry. Then, 10 μL of a suspension of the RuP_2_@N-P-C catalyst (5 mg·mL^−^^1^) in acetone plus nafion was dropped on the GCE and it was allowed to dry at air.

## 3. Results and Discussion

Synthesis and Characterization of RuP_2_@N-P-C

**Scheme 1:**
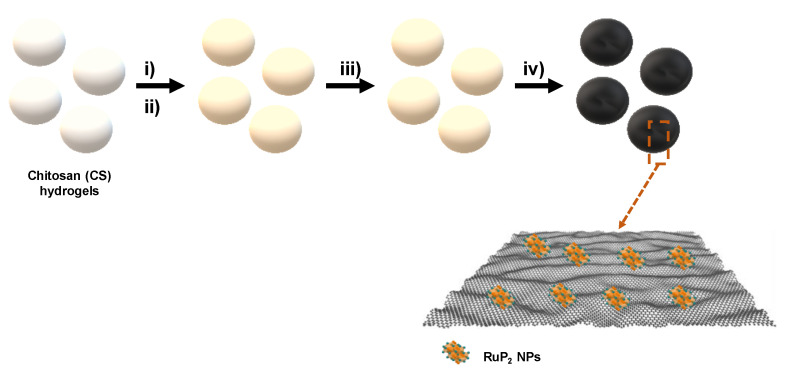
Schematic illustration of the synthesis of RuP_2_@NPC, (**i**) impregnation of CS hydrogels with RuCl_3_ solution; (**ii**) impregnation of Ru containing CS beads with P_2_O_5_; (**iii**) supercritical drying of CS-Ru-Phosphate beads and (**iv**) pyrolysis at 900 °C under inert atmosphere.

RuP_2_ nanoparticles embedded on graphitic porous N-P dual doped graphene were synthesized following a straightforward procedure. Briefly, an acidic chitosan (CS) solution was dropped into a concentrated NaOH solution to form CS microspheres of millimetric size. CS beads were then washed with water to neutralize the pH and the resulting hydrogels were impregnated with a RuCl_3_ solution. CS hydrogels were immersed in RuCl_3_ for 24 h, instead of dissolving CS in the metallic solution as an instantaneous gelation occurs when acetic acid is added to the CS suspension with Ru^3+^, thus, hindering the formation of microspheres in the subsequent steps because of the solution viscosity. Subsequently, the RuCl_3_/CS hydrogels were converted into alcogels by exchanging water with ethanol gradually at increasing ethanol/water ratios (from 10% to 100% ethanol). The as-obtained alcogels were then immersed for 24 h in a P_2_O_5_ solution dissolved in ethanol. Superior adsorption efficiency of phosphate ions into CS matrix is a result of electrostatic interactions of protonated groups of CS and phosphate anions [31]. Drying under supercritical CO_2_ conditions is in general preferred as it preserves the porosity of the beads preventing their shrinkage and collapse of their structure, thus affording light-weight and high surface area aerogels [26,41]. Scheme 1 illustrates the preparation process of the material.

We first proceeded to prepare N,P-dual doped graphitic carbon beads without the addition of metals to see the effect of P doping on the surface area. Upon pyrolysis, highly porous graphitic 3D N,P-dual doped carbons are obtained. Figure S1 shows the structure of the beads obtained upon pyrolysis of CS beads containing phosphate ions. As it can be seen, N,P-dual doped graphitic carbon (N-P-C) contains domains of large porosity similar to KOH-treated carbons obtained from pyrolysis of CS [42,43]. Adding P to CS hydrogels was found to help increase the specific surface area due to the formation of P_2_O_5_ that facilitates the gasification of carbon and distorts the graphitic structure [44]. We found that upon pyrolysis of phosphate-containing CS microspheres, the surface area increases from 241 m^2^∙g^−^^1^ for undoped CS beads to 817 m^2^∙g^−^^1^. The increase observed upon pyrolysis of the beads verifies the role of P_2_O_5_ as both a porogen agent and a dopant. Figure S2 shows the isotherm of N_2_ adsorption-desorption of N-P-C. Figure S3 shows the scanning transmission electron microscopy (STEM) images in bright field mode of N-P-C. The presence of holes in the layered material reveals the presence of a hierarchical porosity. To confirm the presence of the P element in the structure of N-P-C, ^31^P solid-state nuclear magnetic resonance (ss ^31^P NMR) spectroscopy was used. As it can be seen in Figure S4, the presence of a broad peak centered around −4.3 ppm reveals the presence of various polyphosphate species [32,45].

The addition of Ru results in a decrease of the surface area (765 m2∙g−1), however the P amount contained in the material increases. Incorporation of multivalent cations into CS matrix generally leads to enhanced adsorption of phosphate ions explaining the increase in P content in RuP_2_@N-P-C [31]. Table 1 summarizes the chemical composition of RuP_2_@N-P-C and N-P-C as determined by elemental analysis and inductively coupled plasma-optical emission spectroscopy (ICP-OES).

**Figure 1:**
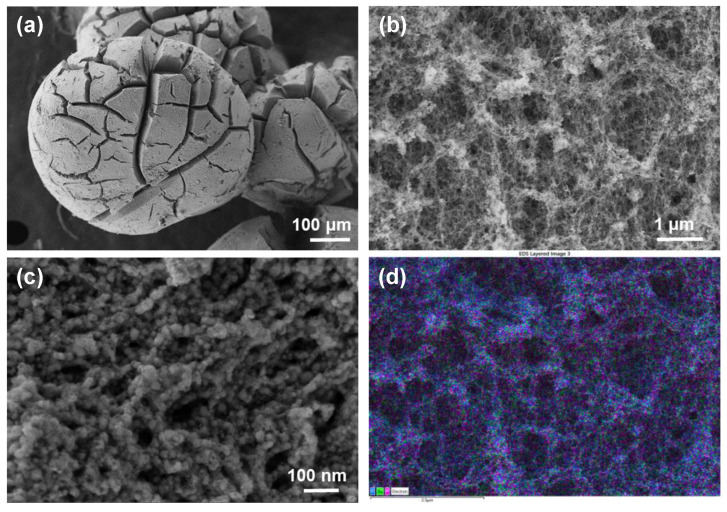
FESEM images (**a**–**c**) of RuP_2_@N-P-C at different magnifications and EDS layered image (**d**) showing the even distribution of C, Ru and P elements.

High-resolution field-emission scanning electron microscopy (HRFESEM) was used to examine the morphology of the as-obtained porous materials. As shown in Figure 1, RuP_2_@N-P-C beads are shaped similarly to a Bristlecone pine. At high magnifications, a fibrillated morphology is revealed (Figure 1c). Associated energy-dispersive X-ray spectroscopy (EDX) images (Figure 1d and Figure S5) reveal that Ru, P and C elements are evenly distributed on the surface.

X-Ray diffraction was used to characterize the crystal structure of RuP_2_@N-P-C. As displayed in the XRD pattern of RuP_2_@NPC (Figure S6), the peaks depicted at 23°, 30.4°, 35°, 36.1°, 38.5°, 39.2°, 46.1°, 47.1°, 47.5°, 49.9°, 50.2°, 54.9°, 56.2°, 56.9°, 59.9°, 65.1°, 68.6°, 72.1°, 73.6°, 74.5° and 76.2° correspond to the (110), (020), (012), (101), (021), (111), (022), (112), (121), (013), (211), (031), (103), (122), (113), (200), (123), (202), (024), (114) and (041) planes of the orthorhombic RuP_2_ phase, respectively [46,47].

Raman spectroscopy was used to assess the graphitization of the carbon obtained upon pyrolysis. Typical peaks observed around 2700, 1595 and 1354 cm^−1^ in the Raman spectrum of RuP_2_@N-P-C (Figure S7) correspond to the 2D, G and D bands, respectively. The I_D_/I_G_ ratio of 0.85 found for RuP_2_@N-P-C indicates the formation of a graphitized carbon instead of amorphous carbon [48,49].

**Figure 2:**
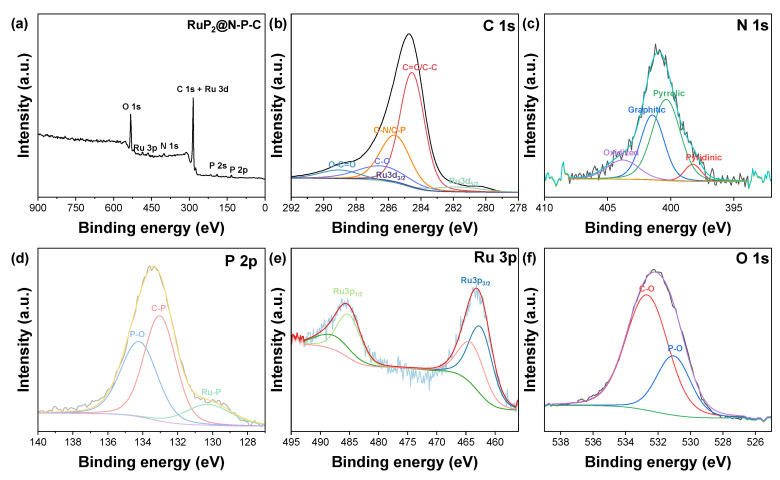
(**a**) XPS survey spectrum of RuP2@N-P-C. High-resolution XPS spectra of (**b**) C 1s, (**c**) N 1s, (**d**) P 2p and (**e**) Ru 3p and (**f**) O 1s.

X-Ray Photoelectron Spectroscopy was conducted to assess the surface chemical state as well as the surface elemental composition of RuP_2_@NPG. XPS analysis of RuP_2_@NPG reveals the presence of C, Ru, N, O and P. Figure 2 shows the high resolution XPS of the different elements present in RuP_2_@NPG. In the C1s spectrum, the peak centered at 284.5 eV corresponds to C=C, while the ones at 285.6, 286.5 and 289.1 eV correspond to C-N/C-P, C-O and O-C=O, respectively [50,51]. Ru3d_5/2_ and Ru3d_3/2_ occur at shifted binding energies than the ones expected at 280.2 eV and 284.4 eV. The experimental N1s peak can be deconvoluted into four components at 398.2, 400.1, 401.4 and 403.7 eV which are attributed to pyridinic N, pyrrolic N, graphitic N and oxidized N, respectively [51,52,53]. The components of the P2p spectrum at 130.1, 133 and 134.2 eV correspond to P-Ru, P-C and P-O, respectively [51,54]. The latter peak might be due to the presence of residual phosphates or from oxidized P-O species resulting from incomplete P reduction or formed during air exposure [52]. In the Ru3p spectrum, the peaks centered at 462.6 and 485.1 eV correspond to Ru3p_3/2_ and Ru3p_1/2_, respectively. The peaks observed at 463.9 and 487.8 eV correspond to oxidized RuP_2_ species due to unavoidable air exposure [53,54]. Regarding oxygen atoms, the experimental O1s peak can be deconvoluted into two components at 531 eV and 532.5 eV which are attributed to P-O and C-O bonds, respectively [45,55]. 

**Figure 3:**
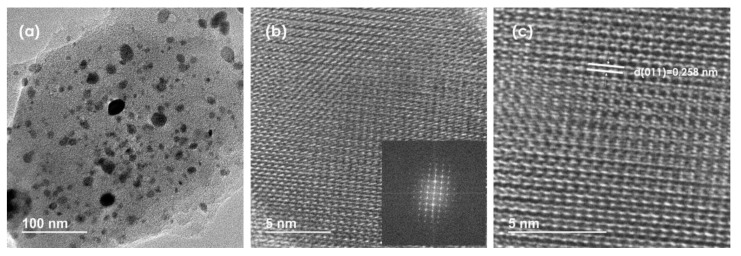
(**a**–**c**) HRTEM images of RuP_2_@N-P-C at different magnifications. The inset in the image (**b**) corresponds to the FFT spectrum of the electron diffraction from one RuP_2_ nanoparticle supported on N-P-C.

High-resolution transmission electron microscopy (HRTEM) images reveal the layered nature of RuP_2_@N-P-C as well as the presence of nanoparticles of different sizes. (Figure 3a). HRTEM was used to further confirm the nature of ruthenium phosphide formed. Figure 3c evidences the presence of lattice fringes with a spacing of 0.258 nm corresponding to the (011) plane of orthorhombic RuP_2_ [47]. Figure S8 shows the EDX spectrum of one of the nanoparticles analyzed. It was found that the elements composing the nanoparticle are Ru and P. Dark-field STEM (DF-STEM) images (Figure S9) reveal that RuP_2_ nanoparticles of different sizes are evenly distributed on the N-P-C support. The average size of the nanoparticles determined by measuring a statistically relevant number of nanoparticles from HRFESEM and DF-STEM images is 8.7 nm ± 4.7 nm.

## 4. Electrochemistry

**Figure 4:**
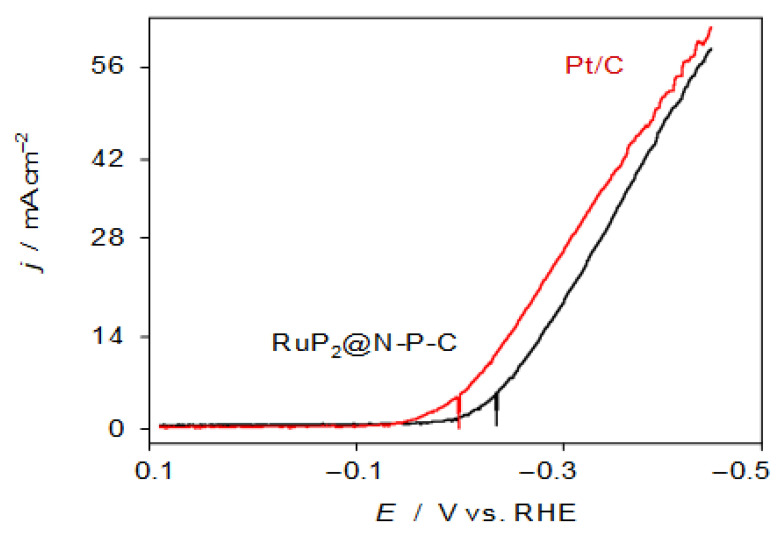
Negative-going LSVs of RuP_2_@N-P-C- (black) and Pt/C-modified (red) glassy carbon electrodes under stirring conditions (650 rpm) in contact with 0.50 M H_2_SO_4_. Potential scan rate 5 mV.s^−1^.

Figure 4 shows the negative-going linear potential scan voltammograms (LSVs) recorded at 5 mV.s^−1^ for GCEs modified with RuP_2_@N-P-C (black) and Pt/C (red) in contact with 0.50 M H_2_SO_4_ solution under stirring. One can see that the catalytic effect of HER exerted by RuP_2_@N-P-C, although comparable to that of 20% Pt/C, does not improve the onset potential. The Tafel analysis of the rising portion of the voltammetric wave, however, yields satisfactory values of the onset potential (−225 ± 5 vs. RHE), Tafel slope (27.1 ± 0.6 mV.decade^−1^), the exchange current ((1.0 ± 0.3) × 10^−2^ mA.cm^−2^), and the overpotential at a current density of 10 mA cm^−2^ (−300 ± 10 mV vs. RHE), when compared with other catalysts (see Table S1) in recent literature [34,35]. In contrast, in contact with 1.0 M KOH the performance of the RuP_2_@N-P-C- catalyst is clearly lower with that of Pt/C, as can be seen in Figure S10. 

The difference in the electrocatalytic performance of RuP_2_@N-P-C in acidic and alkaline media can be rationalized on considering that the initial step (Volmer step) of HER in acidic media,

H^+^ _aq_ + e^−^ + C* → H-C* (1)

involves the adsorption of the generated H atom onto the surface of the catalyst active site (denoted by C* in Equation (1)) [35]. In alkaline media, the concentration of hydrogen ions is drastically diminished and the Volmer step are likely to include a water-dissociation step [36,37],

H_2_O + e^−^ + C* → H-C* + OH^−^_aq_ (2)

Accordingly, in alkaline electrolytes, the cleavage of water O–H bond and the transport of OH^−^ from the catalyst surface to the electrolyte would be part of the limiting step in the overall HER process [38].

Similar considerations can be applied to the subsequent step in the Volmer-Heyrovsky pathway which can be represented as,

H-C* + H^+^ _aq_ + e^−^ → H_2_ + C* (3)

H-C* + H_2_O + e^−^ → H_2_ + C* + OH^−^_aq_ (4)

in acidic (Equation (3)) and alkaline (Equation (4)) media, respectively. In the Volmer-Tafel pathway, however, the second step

H-C* + H-C* → H_2_ + C* (5)

should be minimally influenced by pH changes.

**Figure 5:**
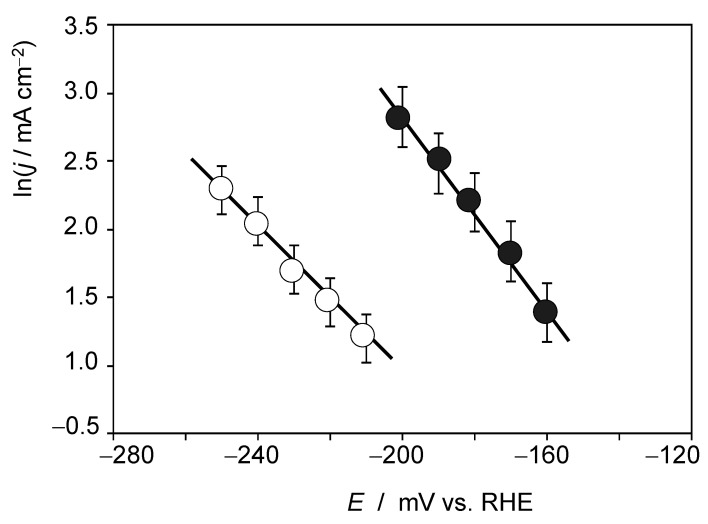
Tafel plots from the foot of the voltammetric curves recorded at RuP_2_@N-P-C-modified GCEs immersed into 0.50 M H_2_SO_4_ + 0.15 M LiClO_4_ (solid circles) and 0.50 M H_2_SO_4_ + 0.03 M LiClO_4_ solutions (circles). Potential scan rate 2 mV.s^−1^. Averaged values from three replicate measurements.

In this context, the importance of interfacial interactions has been emphasized [38] and the impact of alkali ions in HER at Pt and PtNi surfaces has been reported [39,40]. Accordingly, we explored the influence of Li^+^ ions on the catalytic activity of RuP_2_@N-P-C. This effect can be clearly seen under quiescent conditions, as is illustrated in Figure S11. Here, the negative-going LSVs of RuP_2_@N-P-C-modified GCE in contact with 0.50 M H_2_SO_4_ and 0.50 M H_2_SO_4_ + 0.15 M LiClO_4_ solutions are depicted. Although the voltammograms show irregular features due to the formation of H_2_ bubbles at relatively high overpotentials, one can see that the currents are markedly enhanced in the presence of Li^+^ ions in the electrolyte. Figure 5 show the Tafel plots for 0.50 M H_2_SO_4_ + 0.15 M LiClO_4_ and 0.50 M H_2_SO_4_ + 0.03 M LiClO_4_ solutions, in both cases with satisfactory linearity in the ln(j) vs. E plots.

Experimental data at different concentrations of Li^+^ reveal that the Tafel slope and the exchange current density increase with the concentration of alkali metal cation, whereas the onset potential remains essentially unchanged. Pertinent data can be seen in Table S1. Experiments at relatively large potential scan rates produced sigmoidal curves interrupted by the sharp transients associated to the formation of H_2_ bubbles over the electrode surface. This can be seen in Figure S12 where the CV of RuP_2_@N-P-C-modified glassy carbon electrode recorded under quiescent conditions in contact with 0.50 M H_2_SO_4_ + 0.27 M LiClO_4_ solution is presented. Qualitatively, the recorded CV response agrees with that theoretically described for electrocatalytic processes involving species in solution phase for large values of the rate constant k [56]:

(6) $$i=\frac{nFAcD^{1/2}\;k^{1/2}}{1+\exp[\;\frac{nF}{RT}\;\left(E-E_{1/2}\right)}$$

In this equation, c denotes the concentration of the electroactive species, D its diffusion coefficient and E_1/2_ the half-wave potential. At sufficiently high cathodic potentials, the above equation can be simplified to [56,57]:

(7) $$i_{\lim}=nFAcD^{1/2}k^{1/2}$$

i.e., the current tends to a limiting value (*i*_lim_) regardless the potential scan rate. Figure S13 illustrates the variation in the limiting current and the half-wave potential with the concentration of Li^+^ in 0.5 M H_2_SO_4_ solutions.

Although the situation studied here involves a solid catalyst (i.e., differs from the electrocatalysis by species in solution), the above consideration can in principle be taken as a reasonable initial approximation. For our purposes, there are different aspects to emphasize:

(a) The HER process at RuP_2_@N-P-C, a porous material, will involve some restricted diffusion of hydrogen ions in solution through the external region of the solid in order to be adsorbed onto the different active sites. This is supported by abundant literature describing the differences observed between continuous and discrete monolayers and multilayers of catalysts such as MoS_2_ [58].

(b) The diffusion of different ionic species through the layers of porous catalysts can be taken as analogous to that occurring in redox polymers [59,60,61,62] in which co-diffusion effects have to be considered [63,64,65,66]. As a result, different diffusive behaviors can operate.

(c) Co-diffusion effects can be rationalized pm considering that, in solution phase, H^+^ and Li^+^ are (mainly) in the form of H_3_O^+^ and Li(H_2_O)_n_^+^ ions. Then, their penetration into the pores of the solid involves more or less extensive de-hydration. This process, and the subsequent ionic diffusion through the pores of the solid, could be particularly favorable for Li^+^. Accordingly, although H^+^ diffusion is faster Li^+^ diffusion in the electrolyte, the transport of H^+^ through the porous material could be facilitated by the presumably faster diffusion of Li^+^ in the solid.

In this scenario, two extreme diffusive behaviors can be considered for the diffusion through two phases, electrolyte and porous catalyst: full coupling and absence of coupling between phases. The first situation operates at low electrolyte concentrations and the system experiences a Cottrell-type behavior with an effective diffusion coefficient, *D*_eff_, given by:

(8) $$D_{\mathrm{eff}}=\frac{D_{\mathrm{solid}}\;c_{\mathrm{solid}}+D_{\mathrm{electrolyte}}\;c_{\mathrm{electrolyte}}}{c_{solid}+c_{electrolyte}}$$

where *D*_solid_, *D*_electrolyte_, represent the diffusion coefficients of the diffusing species through the solid and the electrolyte and *c*_solid_, *c*_electrolyte_, the respective concentrations. At high electrolyte concentrations and short times (typically shorter than 10–20 ms [59,60]), the flux of the diffusing species will be the sum of the independent diffusive contributions in each phase. Then, the system will display a Cottrell-type behavior characterized by the product between an effective concentration, *c*_eff_, and the effective diffusion coefficient, *D*_eff_, given by the expression:

(9) $$c_{\mathrm{eff}}D_{\mathrm{eff}}=c_{\mathrm{solid}}\;D_{\mathrm{solid}}^{1/2}+c_{\mathrm{electrolyte}}\;D_{\mathrm{electrolyte}}^{1/2}$$

These situations have been studied for microparticulate films of dinuclear alkynyl-diphosphine Au(I) complexes [67] and Prussian blue [68].

The above expressions are analogue to those proposed for the co-diffusion of two electroactive species (A, B) in a unique phase [63,64,65,66]. Coupled diffusion is described by:

(10) $$D_{\mathrm{eff}}=\frac{D_{A}\;c_{A}+D_{B}\;c_{B}}{c_{A}+C_{B}}\;$$

Here, *D*_A_, *D*_B_, represent the diffusion coefficients of the species A and B in the electrolyte and *c*_A_, *c*_B_, their respective concentrations in this phase. When no coupled diffusion occurs,

(11) $$c_{\mathrm{eff}}D_{\mathrm{eff}}^{1/2}=c_{A}D_{A}^{1/2}+c_{B}D_{B}^{1/2}$$

These situations have been experimentally tested for the particular case in which the A and B species are linked by an interconversion reaction [69].

A second effect on HER electrocatalysis associated to the presence of Li^+^ ions in the electrolyte can be considered: the co-adsorption. The effect of OH^−^, H_2_O and cations co-adsorption on Pt surfaces has been studied with regard of HER [70,71,72]. Here, a variety of factors have been balanced (formation of adducts, destabilization of OH_ads_, partial charge retention, etc.), but further research is needed to achieve a complete view of this matter [38].

Qualitatively, these approaches can be applied to explain the accelerating effect of Li^+^ ions on HER at porous catalysts. Focusing our attention to the diffusive effects, and at the expense of the development of a detailed theoretical model, it is pertinent to note that if no coupling diffusion of H^+^ and Li+ ions occurs, no influence of Li^+^ on the HER process should take place. If coupled diffusion occurs, and if the diffusion of Li^+^ is faster than that of H^+^ through the porous film, the effect of Li^+^ would be the increase in the effective (co)diffusion of the system. Assuming that co-diffusion is operative, Equations (6) and (7) can be approximated by:

(12) $$i=\frac{nFA\;{\left(D_{H+}c_{H+}+D_{\mathrm{Li}+}\;c_{\mathrm{Li}+}\right)}^{1/2}\;k^{1/2}}{1+\exp(\frac{nF}{RT}\;\left(E-E_{1/2}\right)}$$

(13) $$i_{\lim}=nFA\;{\left(D_{H+}c_{H+}+D_{\mathrm{Li}+}c_{\mathrm{Li}+}\right)}^{1/2}k^{1/2}\;\;\;$$

where *D*_H+,_ *D*_Li+_ are the diffusion coefficients of H^+^ and Li^+^ and *c*_H+_, *c*_Li+_ their respective concentrations in the electrolyte. Then, in experiments performed varying the concentration of Li^+^ at a constant concentration of hydrogen ions such as in Figure S13, taking *i*º_lim_ = *nFAD*_H+_^1/2^*c*_H+_k^1/2^, we obtain,

(14) $$f\left(i_{\lim}\right)=\frac{i_{\lim}-i_{\lim}^{{}^{\circ}}}{i_{\lim}^{{}^{\circ}}}=\frac{{\left(D_{H+}c_{H+}+D_{\mathrm{Li}+}c_{\mathrm{Li}+}\right)}^{1/2}}{{\left(D_{H+}c_{H+}\right)}^{1/2}}\;$$

**Figure 6:**
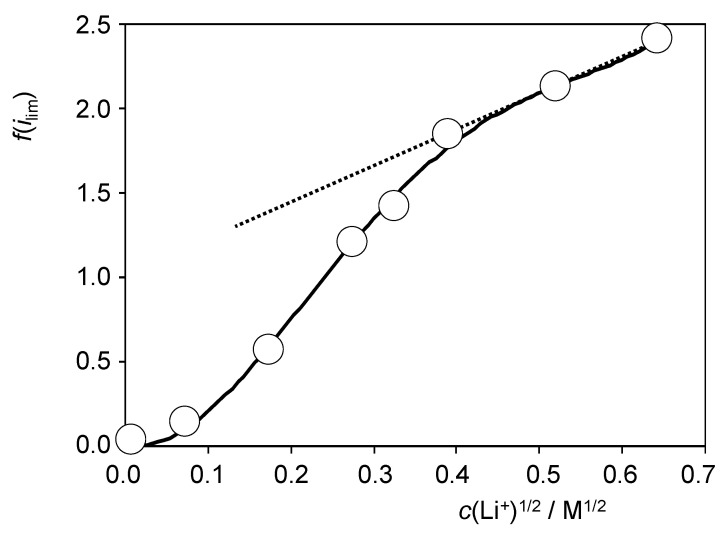
Variation in the f(*i*_lim_) ratio with the square root of the Li^+^ concentration in CVs at RuP_2_@N-P-C-modified GCEs recorded under quiescent conditions in contact with 0.50 M H_2_SO_4_ + LiClO_4_ solutions in conditions such as those in Figure S12. The continuous line corresponds to the polynomial fit of the entire set of data while the dotted line defines the straight line fitting for the values at high concentrations of Li^+^.

At large Li^+^ concentrations, Equation (14) tends to a linear variation in f(*i*_lim_) on the square root of the concentration of Li^+^ in the electrolyte. Figure 6 shows the variation in the f(*i*_lim_) ratio with the square root of the Li^+^ concentration resulting from voltammetric data such as depicted in Figure S12. One can see that the values at high Li^+^ concentrations can reasonably be fitted to a straight line. The slope of the linear plot (2.30 ± 0.05) permits to evaluate a (*D*_Li+_/*D*_H+_)^1/2^ ratio of 4.6. This result implies that the diffusion is faster for Li^+^ than for H^+^, an effect that can be understood assuming that the rate-determining step of the diffusional process is that occurring through the porous phosphide material. In short, this means that the porous material can accommodate more efficiently Li^+^ ions than adsorb hydrogen ions. At this stage, our results suggest that, despite the approximate character of the previous theoretical considerations, at least a part of the contribution of Li^+^ ions to promote HER at RuP_2_@N-P-C-modified GCEs can be attributed to co-diffusion effects. A more detailed understanding of Li^+^ effect obviously requires further research to elucidate influence of adsorption and ion trapping processes.

**Figure 7:**
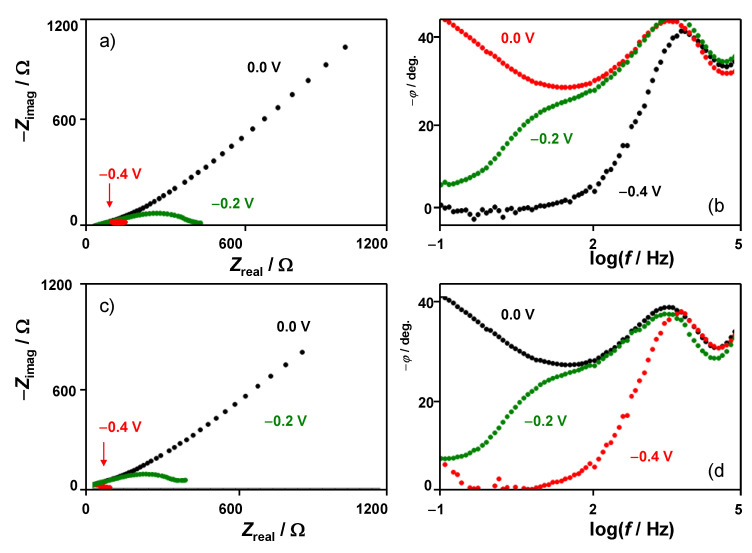
Impedance spectra of RuP_2_@N-P-C-modified GCEs in contact with (**a**,**b**) 0.50 M H_2_SO_4_ and (**c**,**d**) 0.50 M H_2_SO_4_ + 0.14 M LiClO_4_ solutions at different bias potentials. (**a**,**c**) Nyquist plots of *Z*_real_ vs. −*Z*_imag_; (**b**,**d**) Bode plots of −*ϕ* vs. log*f*.

EIS data were consistent with the foregoing set of considerations. When bias potentials separated from the proton discharge are applied, light differences were obtained RuP_2_@N-P-C-modified GCEs immersed into 0.50 M H_2_SO_4_ and 0.50 M H_2_SO_4_ + 0.14 M LiClO_4_ solutions (Figure 7 and Supplementary Information, Figure S14). However, when the bias potential approaches those where the HER starts, the presence of Li^+^ ions in the electrolyte determines a decrease in the total impedance values at low frequencies. The Nyquist plots show two overlapping capacitive loops which can be fitted to the equivalent circuit shown in Figure S15. Remarkably, the negative increase in the bias potential does not alter significantly the high-frequency loop but determines important changes in the low-frequency region where the capacitive effects tend to vanish on increasing the hydrogen evolution. The charge transfer resistance values significantly decrease, and this decrease is larger as the Li^+^ concentration is, as can be seen in Figure S16 of supplementary Information for different bias potentials.

## 5. Conclusions

In this contribution, simultaneous P-doping and growth of crystalline ruthenium diphosphide was achieved through a straightforward approach consisting on the pyrolysis of Ru^3+^-CS beads on which phosphate anions were previously co-adsorbed. Upon pyrolysis, highly porous N,P-dual-doped defective graphitic carbon on which crystalline RuP_2_ nanoparticles are embedded was obtained. RuP_2_@N-P-C films deposited onto GCEs exert an electrocatalytic effect on HER in acidic media. This effect is significantly increased upon addition of Li^+^ ions to the electrolyte. Voltammetric experiments at variable concentrations of this alkali cation suggest that co-diffusion effects, Li^+^ diffusing faster than hydrogen ions through the porous catalyst can be responsible, at least partially, for this effect. The sustainable procedure proposed could be applied to prepare other non-precious metal phosphides supported on dual-doped highly porous graphitic carbons. Future studies should rely on the use of in situ/operando techniques [73] to further elucidate the dynamic changes occurring in the electrocatalysts during HER.

## Figures and Tables

**Table 1:** Chemical composition and surface area of N-P-C and RuP_2_@N-P-C.

Sample	C (%)	N (%)	P (%)	Ru (%)	H (%)	Surface Area (m^2^/g)
N-P-C	59.6	1.9	3.1	-	0.5	817
RuP_2_@N-P-C	53.1	2	8.9	6.7	0.78	765

## Data Availability

Not applicable.

## Supplementary Materials

The following supporting information can be downloaded at: https://www.mdpi.com/article/10.3390/nano12203597/s1, Figure S1. FESEM images of N,P-dual doped porous graphitic carbon (N-P-C). Figure S2. N_2_ adsorption-desorption isotherm of N-P-C. Figure S3. Bright Field STEM images of N-P-C. Figure S4. ^31^P SS NMR spectrum of N-P-C. Figure S5. FESEM image and associated EDX mapping of RuP_2_@N-P-C. Figure S6. XRD pattern of RuP_2_@N-P-C. Figure S7. Raman spectrum of RuP_2_@N-P-C. Figure S8. EDX spectrum of RuP_2_@N-P-C. Figure S9. Dark-field STEM images of RuP_2_@N-P-C. Figure S10. Negative-going LSVs of RuP_2_@N-P-C (red) and Pt/C-modified (black) glassy carbon electrodes under stirring conditions (650 rpm) in contact with 1.0 M KOH. Potential scan rate 5 mV.s^−1^. Figure S11. Negative-going LSVs of RuP_2_@N-P-C modified GCE in contact with 0.50 M H_2_SO_4_ (red) and 0.50 M H_2_SO_4_ + 0.15 M LiClO_4_ under quiescent conditions. Potential scan rate 2 mV.s^−1^. Figure S12. CV of RuP_2_@N-P-C-modified glassy carbon electrode recorded under quiescent conditions in contact with 0.50 M H_2_SO_4_ + 0.27 M LiClO_4_ solution. Potential scan initiated at 0.30 V vs. RHE in the negative direction; potential scan rate 50 mV.s^−1^. The arrows mark the ascending (initial cathodic scan) and descending (subsequent positive-going scan) branches of the CV. Figure S13. Variation in: (a) the limiting current and (b) the half-wave potential with the concentration of Li^+^ in CVs at RuP_2_@N-P-C-modified GCEs recorded under quiescent conditions in contact with 0.50 M H_2_SO_4_ + LiClO_4_ solutions. Potential scan initiated at 0.30 V vs. RHE in the negative direction; potential scan rate 50 mV.s^−1^. Figure S14. Nyquist plots of RuP_2_@N-P-C-modified GCEs recorded under quiescent conditions in contact with 0.50 M H_2_SO_4_ and 0.50 M H_2_SO_4_ + 0.14 M LiClO_4_ solutions. Bias potential 0.40 V vs. RHE. The inset shows the magnified view of the high frequency region. Figure S15. Nyquist plots of RuP_2_@N-P-C-modified GCEs recorded under quiescent conditions in contact with a) 0.50 M H_2_SO_4_ and b) 0.50 M H_2_SO_4_ + 0.14 M LiClO_4_ solutions. Bias potential 0.20 V vs. RHE. Experimental data points (black circles) are superimposed to the theoretical impedance spectra (continuous lines) based on the fit of experimental data to the equivalent circuit in c). This equivalent circuit contains the solution resistance (Rs) in series with two parallel RC units, the first one contains the charge transfer resistance (Rct) and the double-layer capacitance (Cdl); the second can be associated to the porous RuP_2_@N-P-C modifier. Its impedance is constituted by a porous resistance (Rpor) and a non-ideal capacitance element, represented by a constant phase element (Qpor). Table S1. Comparison of the catalytic parameters of RuP_2_@N-P-C with other HER catalysts. Figure S16. Variation in the R_por_ determined after fitting the experimental impedance spectra recorded at RuP_2_@N-P-C-modified GCEs in contact with 0.50 M H_2_SO_4_ plus LiClO_4_ solutions with the Li^+^ concentration at different bias potentials.
