# Supplementary material for: Phosphorus-Rich Ruthenium Phosphide Embedded on a 3D Porous Dual-Doped Graphitic Carbon for Hydrogen Evolution Reaction
Source: Nanomaterials (Basel). 2022 Oct 13;12(20):3597. doi: 10.3390/nano12203597 (PMC9606981; doi:10.3390/nano12203597)
Supplement: Supplementary file 1 [file nanomaterials-12-03597-s001.zip › nanomaterials-1951849-supplementary.pdf]

nanomaterials

Supplementary Materials

Phosphorus-Rich Ruthenium Phosphide Embedded on a 3D Porous Dual-Doped Graphitic Carbon for Hydrogen Evolution Reaction

Aicha Anouar [1,2,*], Antonio Doménech-Carbó [3,*] and Hermenegildo Garcia [2,*]

[1] Engineering Division, Euromed Research Institute, EuroMed University of Fes (UEMF), Route de Meknes, Rond-Point de Bensouda, Fès 30070, Morocco
[2] Departamento de Química (UPV), Instituto de Tecnología Química (CSIC-UPV), Universitat Politècnica de València, Av. de los Naranjos s/n, 46022 Valencia, Spain
[3] Departament de Química Analítica, Universitat de València, Dr. Moliner 50, Burjassot, 46100 Valencia, Spain
* Correspondence: a.anouar@ueuromed.org (A.A.); antonio.domenech@uv.es (A.D.-C.); hgarcia@qim.upv.es (H.G.)

Figure S1. FESEM images of N,P-dual doped porous graphitic carbon (N-P-C)
Figure S2. N_2 adsorption desorption isotherm of N-P-C
Figure S3. Bright Field STEM images of N-P-C
Figure S4. Ss ^{31}P NMR spectrum of N-P-C
Figure S5. FESEM image and associated EDX mapping of RuP_2@N-P-C
Figure S6. XRD pattern of RuP_2@N-P-C
Figure S7. Raman spectrum of RuP_2@N-P-C
Figure S8. EDX spectrum of RuP_2@N-P-C
Figure S9. Dark-field STEM images of RuP_2@N-P-C
Figure S10. Negative-going LSVs of RuP_2@N-P-C (red) and Pt/C-modified (black) glassy carbon electrodes under stirring conditions (650 rpm) in contact with 1.0 M KOH. Potential scan rate 5 mV s^{-1}.
Figure S11. Negative-going LSVs of RuP_2@N-P-C modified GCE in contact with 0.50 M H_2SO_4 (red) and 0.50 M H_2SO_4 + 0.15 M $LiClO_4$ under quiescent conditions. Potential scan rate 2 mV.s^{-1}.
Figure S12. CV of RuP_2@N-P-C-modified glassy carbon electrode recorded under quiescent conditions in contact with 0.50 M H_2SO_4 + 0.27 M $LiClO_4$ solution. Potential scan initiated at 0.30 V vs. RHE in the negative direction; potential scan rate 50 mV.s^{-1}. The arrows mark the ascending (initial cathodic scan) and descending (subsequent positive-going scan) branches of the CV.
Figure S13. Variation of: a) the limiting current and b) the half-wave potential with the concentration of Li^+ in CVs at RuP_2@N-P-C-modified GCEs recorded under quiescent conditions in contact with 0.50 M H_2SO_4 + $LiClO_4$ solutions. Potential scan initiated at 0.30 V vs. RHE in the negative direction; potential scan rate 50 mV.s^{-1}.
Figure S14. Nyquist plots of RuP_2@N-P-C-modified GCEs recorded under quiescent conditions in contact with 0.50 M H_2SO_4 and 0.50 M H_2SO_4 + 0.14 M $LiClO_4$ solutions. Bias potential 0.40 V vs. RHE. The inset shows the magnified view of the high frequency region.
Figure S15. Nyquist plots of RuP_2@N-P-C-modified GCEs recorded under quiescent conditions in contact with a) 0.50 M H_2SO_4 and b) 0.50 M H_2SO_4 + 0.14 M $LiClO_4$ solutions. Bias potential –0.20 V vs. RHE. Experimental data points (black circles) are superimposed to the theoretical impedance spectra (continuous lines) based on the fit of experimental data to the equivalent circuit in c). This equivalent circuit contains the solution resistance (R_s) in series with two parallel RC units, the first one contains the charge transfer resistance (R_{ct}) and the double-layer capacitance (C_{dl}); the second can be associated to the porous

RuP2@N-P-C modifier. Its impedance is constituted by a porous resistance (R_{por}) and a non-ideal capacitance element, represented by a constant phase element (Q_{por}).

Figure S16. Variation of the R_{por} determined after fitting the experimental impedance spectra recorded at RuP2@N-P-C-modified GCEs in contact with 0.50 M H_2SO_4 plus $LiClO_4$ solutions with the Li^+ concentration at different bias potentials.

Table S1. Comparison of the catalytic parameters of RuP2@N-P-C with other HER catalysts. Data in this study were taken from LSVs carried out at a potential scan rate of 2 mV s^{-1}.

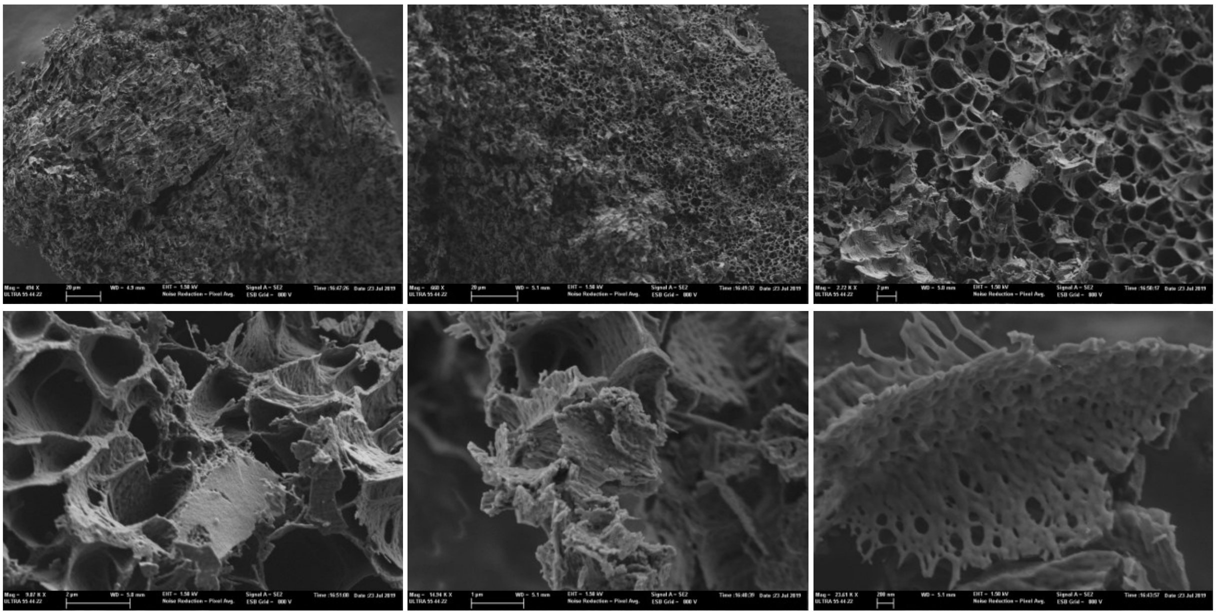

Figure S1. FESEM images of N,P-dual doped porous graphitic carbon (N-P-C).

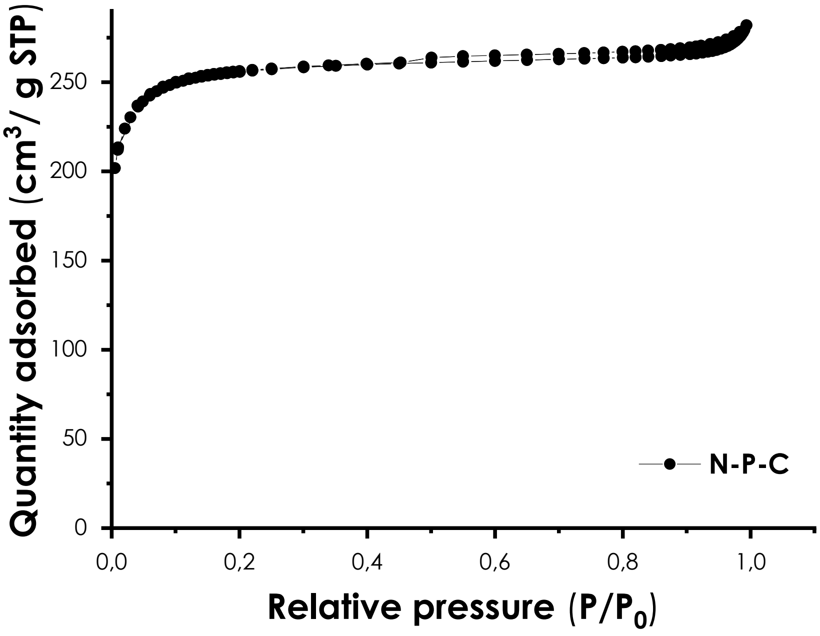

Figure S2. N_2 adsorption desorption isotherm of N-P-C.

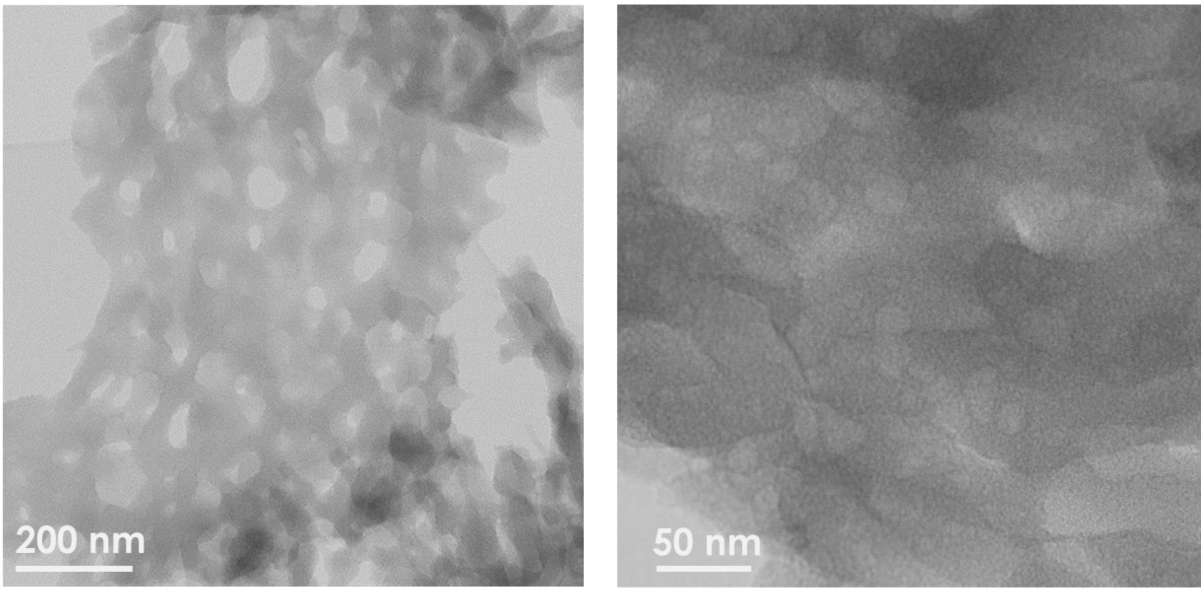

Figure S3. Bright Field STEM images of N-P-C.

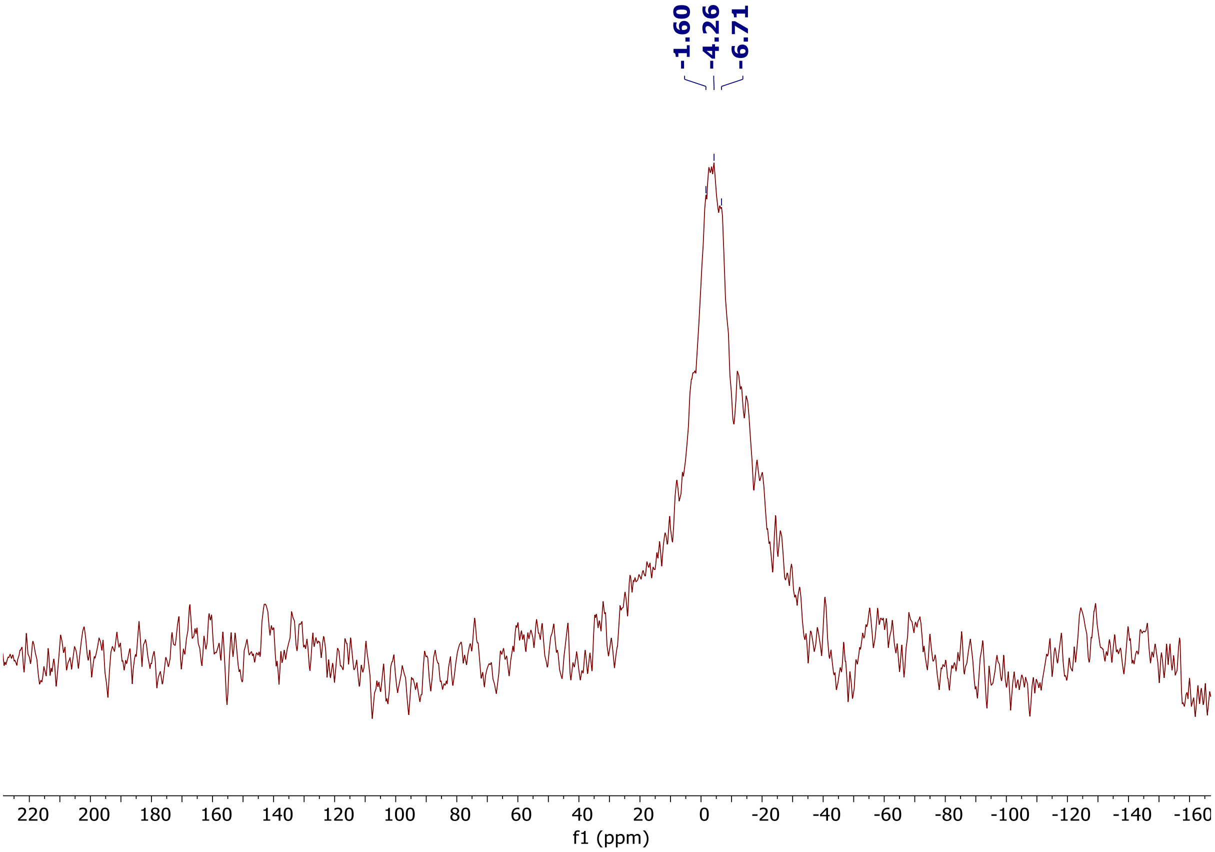

Figure S4. Ss ^{31}P NMR spectrum of N-P-C.

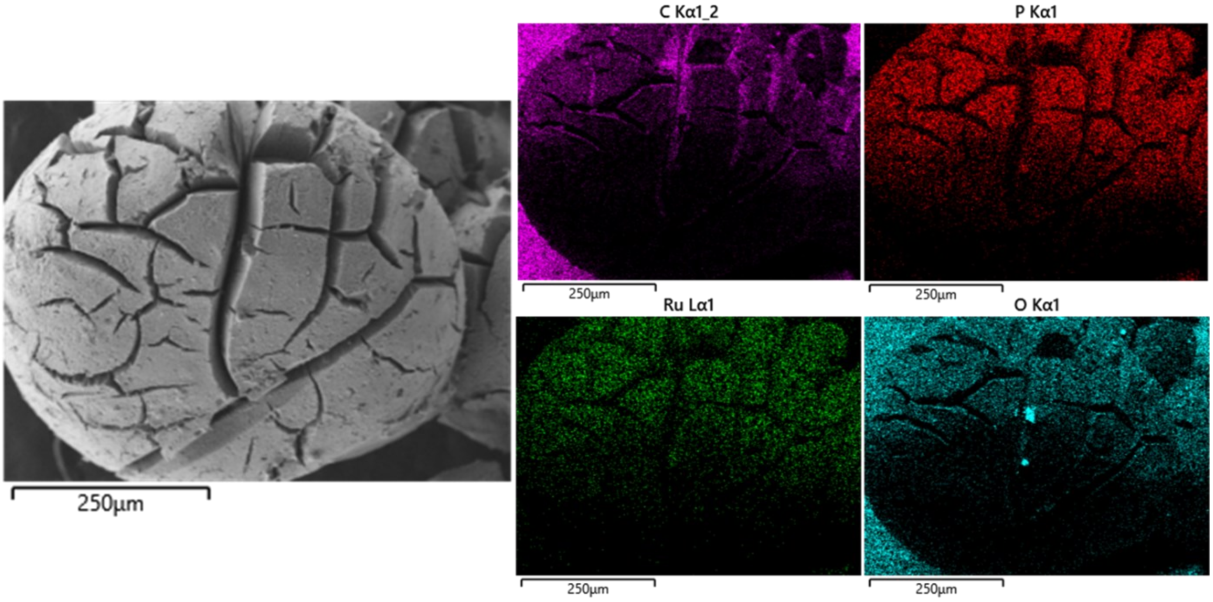

Figure S5. FESEM image and associated EDX mapping of RuP_2@N-P-C.

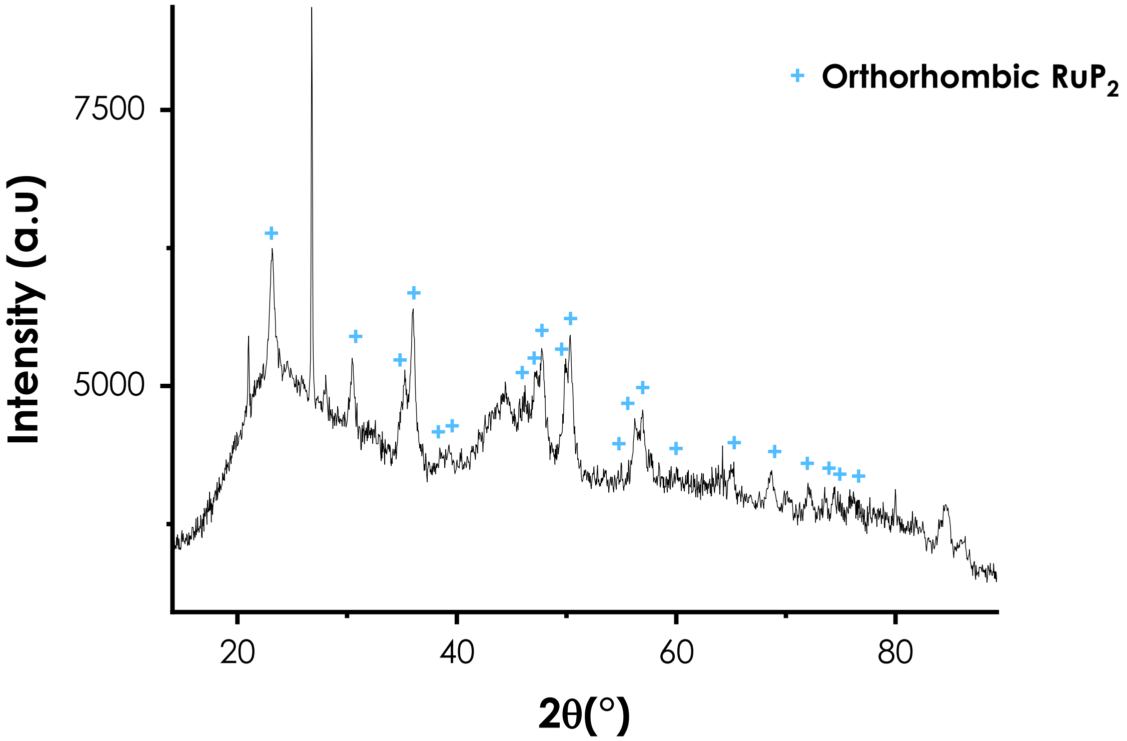

Figure S6. XRD pattern of RuP_2@N-P-C.

As displayed in the XRD pattern of RuP_2@N-P-C, the peaks depicted at 23°, 30.4°, 35°, 36.1°, 38.5°, 39.2°, 46.1°, 47.1°, 47.5°, 49.9°, 50.2°, 54.9°, 56.2°, 56.9°, 59.9°, 65.1°, 68.6°, 72.1°, 73.6°, 74.5° and 76.2° correspond respectively to the (110), (020), (012), (101), (021), (111), (022), (112), (121), (013), (211), (031), (103), (122), (113), (200), (123), (202), (024), (114) and (041) planes of the orthorhombic RuP_2 phase.

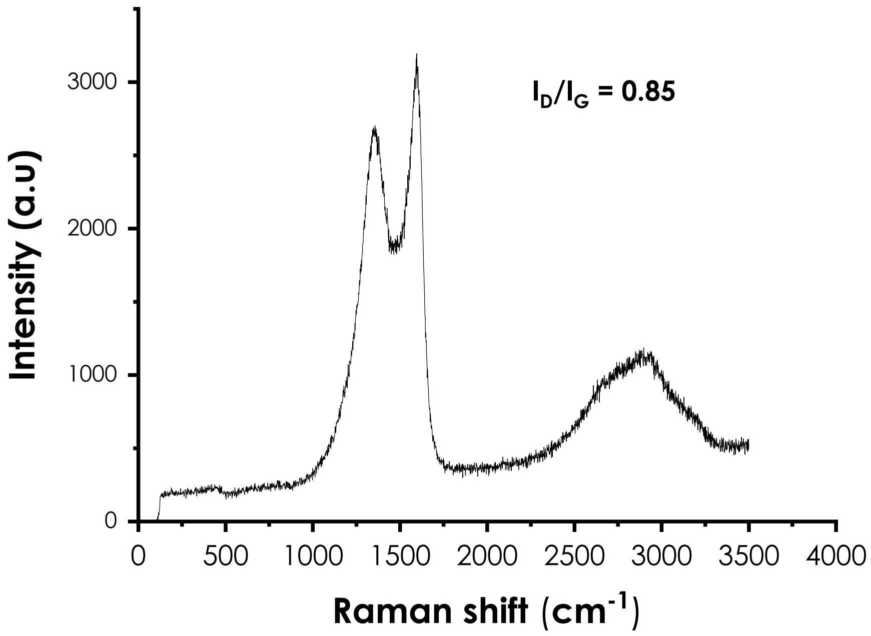

Figure S7. Raman spectrum of RuP_2@N-P-C.

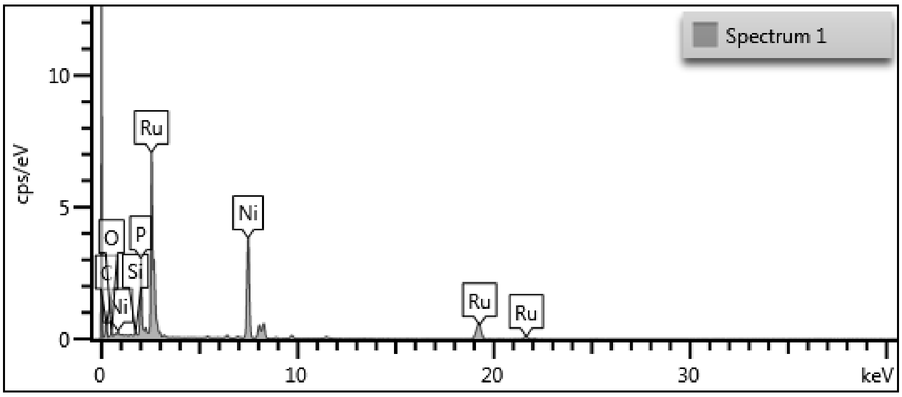

Figure S8. EDX spectrum of RuP_2@N-P-C.

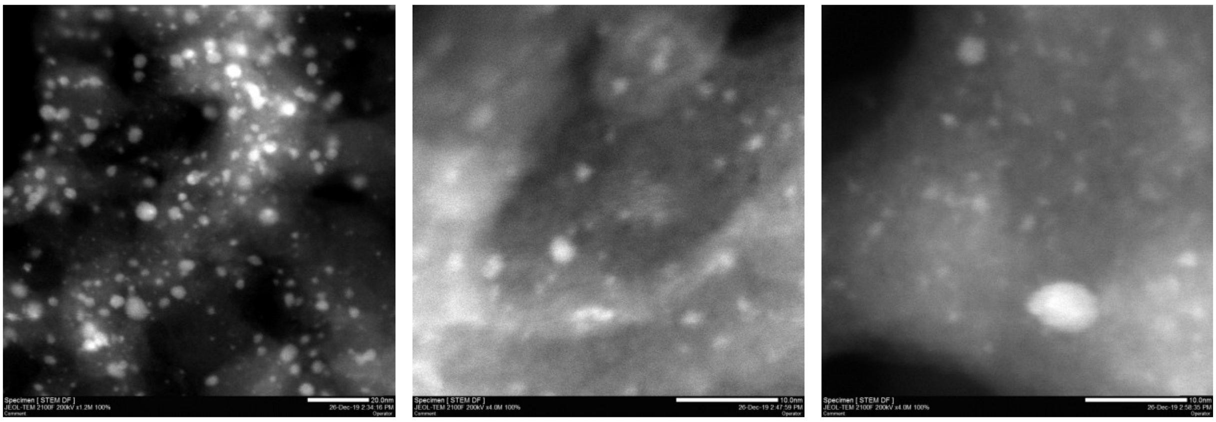

Figure S9. Dark-field STEM images of RuP_2@N-P-C.

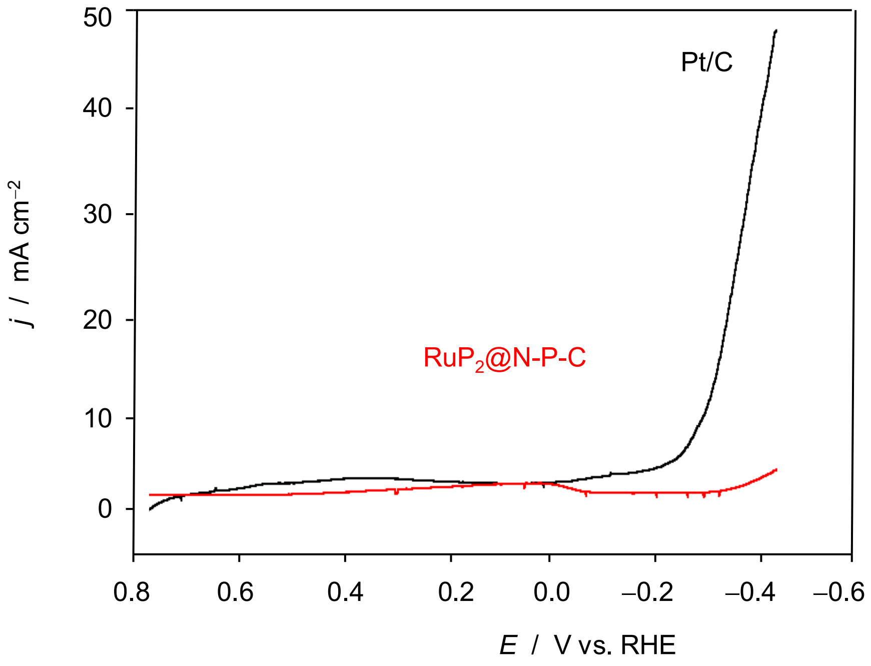

Figure S10. Negative-going LSVs of RuP_2@N-P-C (red) and Pt/C-modified (black) glassy carbon electrodes under stirring conditions (650 rpm) in contact with 1.0 M KOH. Potential scan rate 5 mV s^{-1}.

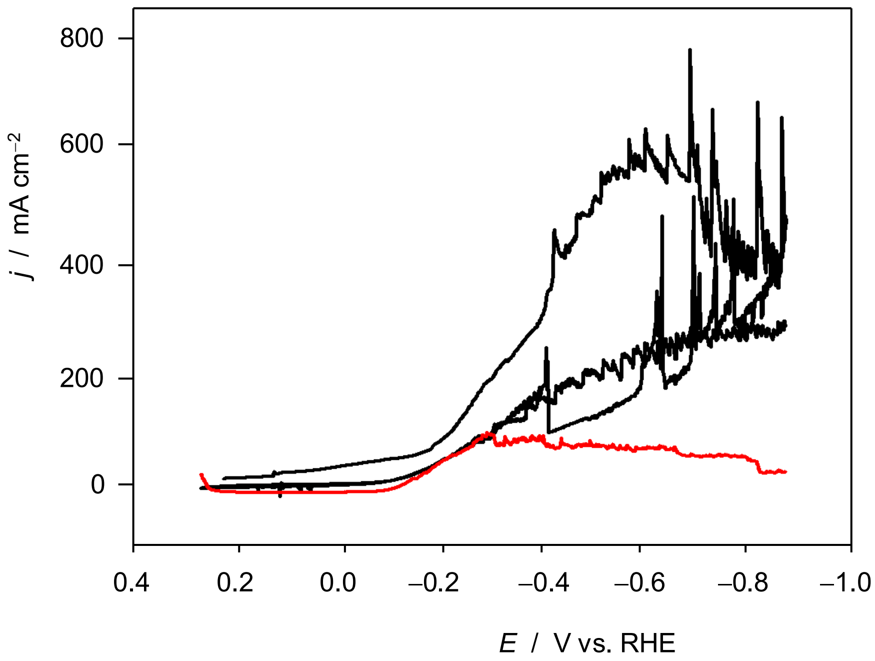

Figure S11. Negative-going LSVs of RuP_2@N-P-C modified GCE in contact with 0.50 M H_2SO_4 (red) and 0.50 M H_2SO_4 + 0.15 M $LiClO_4$ under quiescent conditions. Potential scan rate 2 mV.s^{-1}.

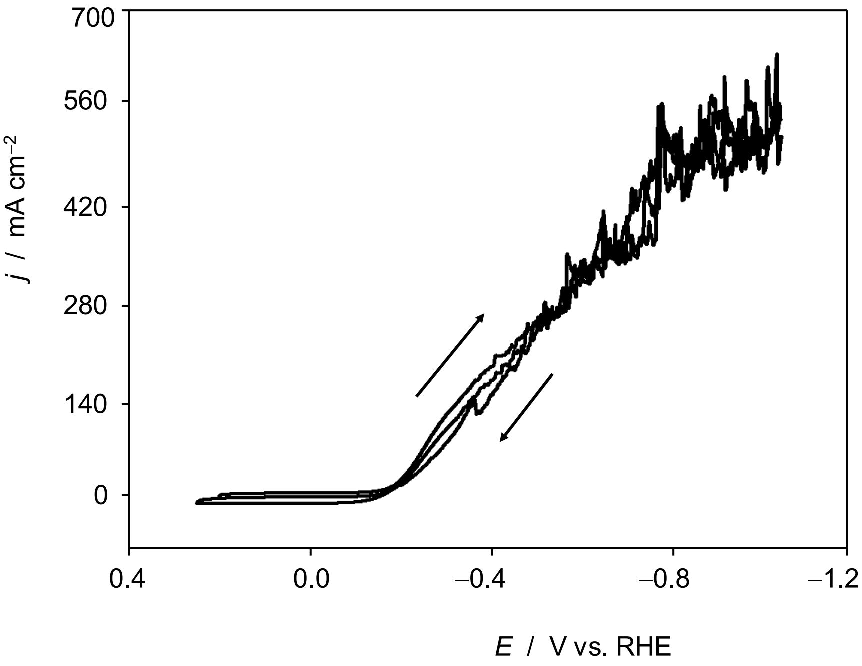

Figure S12. CV of RuP2@N-P-C-modified glassy carbon electrode recorded under quiescent conditions in contact with 0.50 M H_2SO_4 + 0.27 M $LiClO_4$ solution. Potential scan initiated at 0.30 V vs. RHE in the negative direction; potential scan rate 50 mV.s^{-1}. The arrows mark the ascending (initial cathodic scan) and descending (subsequent positive-going scan) branches of the CV.

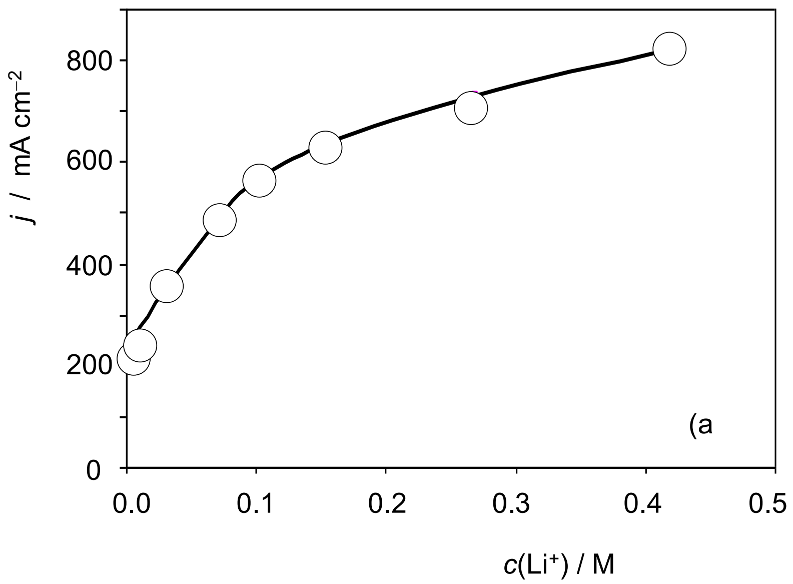

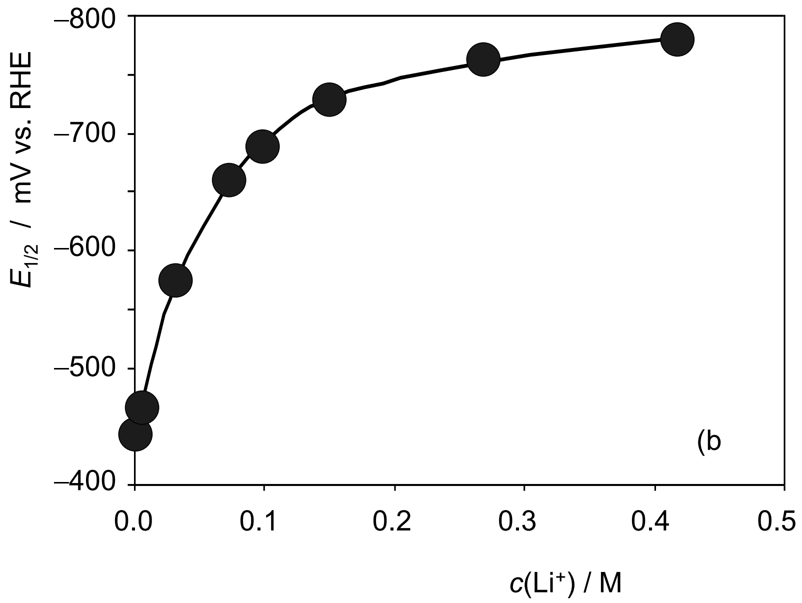

Figure S13. Variation of: (**a**) the limiting current and (**b**) the half-wave potential with the concentration of Li^+ in CVs at RuP_2@N-P-C-modified GCEs recorded under quiescent conditions in contact with 0.50 M H_2SO_4 + $LiClO_4$ solutions. Potential scan initiated at 0.30 V vs. RHE in the negative direction; potential scan rate 50 mV.s^{-1}.

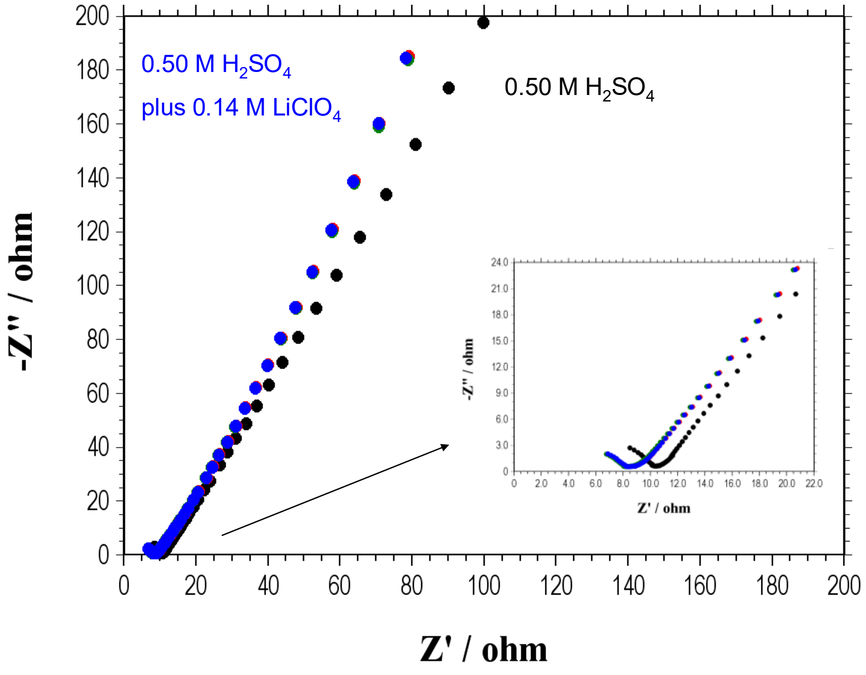

Figure S14. Nyquist plots of RuP_2@N-P-C-modified GCEs recorded under quiescent conditions in contact with 0.50 M H_2SO_4 and 0.50 M H_2SO_4 + 0.14 M $LiClO_4$ solutions. Bias potential 0.40 V vs. RHE. The inset shows the magnified view of the high frequency region.

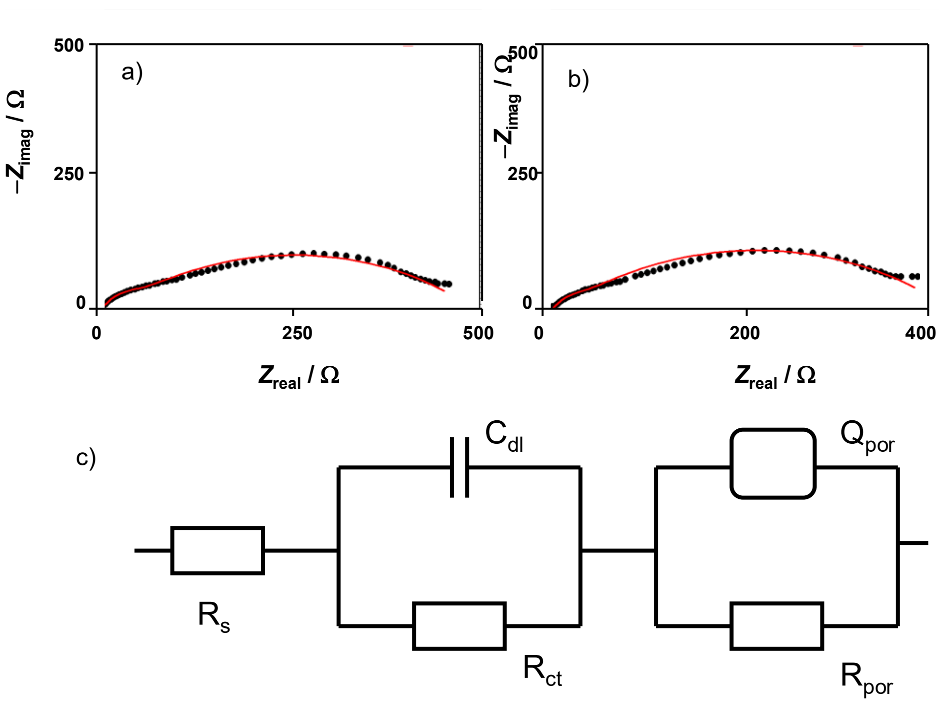

Figure S15. Nyquist plots of RuP_2@N-P-C-modified GCEs recorded under quiescent conditions in contact with (**a**) 0.50 M H_2SO_4 and (**b**) 0.50 M H_2SO_4 + 0.14 M $LiClO_4$ solutions. Bias potential –0.20 V vs. RHE. Experimental data points (black circles) are superimposed to the theoretical impedance spectra (continuous lines) based on the fit of experimental data to the equivalent circuit in (**c**). This equivalent circuit contains the solution resistance (R_s) in series with two parallel RC units, the first one contains the charge transfer resistance (R_{ct}) and the double-layer capacitance (C_{dl}); the second can be associated to the porous RuP_2@N-P-C modifier. Its impedance is constituted by a porous resistance (R_{por}) and a non-ideal capacitance element, represented by a constant phase element (Q_{por}).

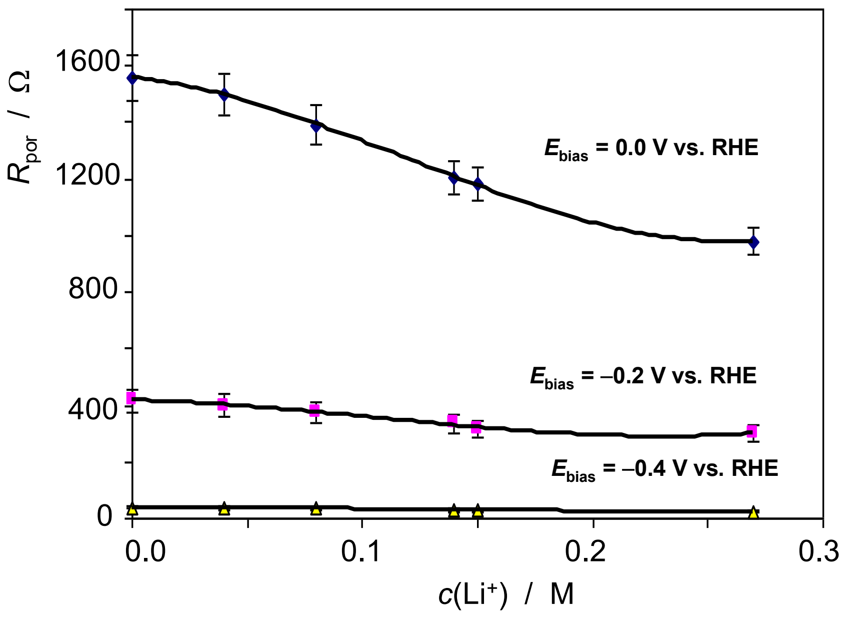

Figure S16. Variation of the R_{por} determined after fitting the experimental impedance spectra recorded at RuP_2@N-P-C-modified GCEs in contact with 0.50 M H_2SO_4 plus $LiClO_4$ solutions with the Li^+ concentration at different bias potentials.

Table S1. Comparison of the catalytic parameters of RuP_2@N-P-C with other HER catalysts. Data in this study were taken from LSVs carried out at a potential scan rate of 2 mV s^{-1}.

**Catalyst**	**Electrolyte**	**E_{onset} (mV vs. RHE)**	**Tafel Slope (mV Decade^{-1})**	**Exchange Current Density (mA cm^{-2})**
Pt/C	0.50 M H_2SO_4	0	30	7.1×10^{-1}
MoS_2	0.50 M H_2SO_4	−237	101	9.1×10^{-4}
RuP_2@N-P-C	0.50 M H_2SO_4	−225 ± 5	27.1 ± 0.6	$(1.0 \pm 0.3) \times 10^{-2}$
RuP_2@N-P-C	0.50 M H_2SO_4 + 0.03 M $LiClO_4$	−235 ± 5	27.2 ± 1.0	$(1.1 \pm 0.3) \times 10^{-2}$
RuP_2@N-P-C	0.50 M H_2SO_4 + 0.15 M $LiClO_4$	−235 ± 5	30.4 ± 0.9	$(1.4 \pm 0.4) \times 10^{-2}$
RuP_2@N-P-C	0.50 M H_2SO_4 + 0.27 M $LiClO_4$	−235 ± 5	30.5 ± 0.9	$(1.6 \pm 0.4) \times 10^{-2}$
